# Involvement of chromosome 6 in endometrial cancer.

**DOI:** 10.1038/bjc.1997.312

**Published:** 1997

**Authors:** M. G. Tibiletti, B. Bernasconi, M. Taborelli, D. Furlan, A. Fabbri, M. Franchi, R. Taramelli, M. Trubia, C. Capella

**Affiliations:** Ospedale di Circolo, Varese and Department of Clinical and Biological Sciences, University of Pavia at Varese, Italy.

## Abstract

**Images:**


British Journal of Cancer (1997) 75(12), 1831–1835
© 1997 Cancer Research Campaign

### Involvement of chromosome 6 in endometrial cancer

**MG Tibiletti[1], B Bernasconi[1], M Taborelli[1], D Furlan[1], A Fabbri[1], M Franchi[2], R Taramelli[3], M Trubia[4] and C Capella[1]**

[1]Ospedale di Circolo, Varese and Department of Clinical and Biological Sciences, University of Pavia at Varese; [2]Obstetric and Gynecological Department, University of Pavia at Varese; [3]Department of Animal Biology, University of Catania; [4]Human Genetics Laboratory S. Raffaele Hospital, Milan, Italy

**Summary** Cytogenetic investigation was performed on direct preparations of 15 endometrial cancers showing different histotypes. Clonal abnormalities were found in 11 out of 13 analysable cases. The modal chromosome number was near diploid in all cases. The abnormal karyotypes contained relatively simple numerical or structural aberrations in the majority of tumours. In contrast, two neoplasms with serous papillary and mixed müllerian morphological features shared multiple complex changes as well as cytogenetic evidence of intratumoral heterogeneity. The most frequent chromosome abnormality in our series of endometrial neoplasms was 6q deletion, which was detected in serous papillary, endometrioid and mixed müllerian tumours. The loss of the 6q region, which is also frequently involved in ovarian carcinoma, suggests a relationship between endometrial and ovarian cancers based on a common histogenesis.

**Keywords:** endometrial tumour; cytogenetic; chromosome 6; yeast artificial chromosome clone

Endometrial cancer is the most common gynaecological malignancy in Italy, accounting for 42% of female genital tract cancers diagnosed from 1983 to 1987 as reported in the Varese Cancer Register (Zanetti and Crosignani, 1992).

The pathogenesis of endometrial carcinoma is heterogeneous, and two different clinical entities referred to as type I and type II can be distinguished (Kurman et al, 1994). Type I is more frequent in younger women and often associated with unopposed oestrogen exposure. It is histologically endometrioid and usually well differentiated, of moderate aggressiveness and frequently preceded by well-defined precancerous lesions (Bokhman, 1983; Smith and McCartney, 1985). Type II cancer includes serous papillary, clear cell and undifferentiated carcinomas that are not associated with well-identified precursor lesions. It is rarer and clinically more aggressive than type I cancer and is usually diagnosed in older women without a history of oestrogen exposure.

In addition to these two types of endometrial cancer, the malignant mixed müllerian tumour can be considered as a rare, but very aggressive, neoplasm.

Until now, little has been known about the process of tumorigenesis of the two main types of endometrial carcinoma. Cytogenetic and molecular genetic studies can provide information that may be relevant for the pathogenesis and may contribute to the identification of specific types of tumour with different biological behaviour. To date, there have been relatively few studies on clonal cytogenetic abnormalities in endometrial cancers. Mitelman (1994) reported 50 cases with clonal chromosome aberrations. More recently, Bardi et al (1995), in a cytogenetic study of 13 endometrial carcinomas, showed that trisomy or tetrasomy 1, trisomy 2, 7, 10 and 12, and loss of chromosome 22 were common alterations.

We have studied chromosome constitutions of 15 endometrial carcinomas (11 of type I, three of type II and one mixed müllerian tumour) using cytogenetic analysis and fluorescence in situ hybridization (FISH) in order to identify the various patterns of chromosome abnormalities and their relationship with different histological types.

*Received 15 January 1996*
*Revised 24 October 1996*
*Accepted 8 January 1997*

*Correspondence to:* MG Tibiletti, Servizio di Anatomia Patologica, Ospedale di Circolo, Viale Borri, 57, 21 100 Varese, Italy

### MATERIALS AND METHODS

Fifteen endometrial carcinomas surgically resected at Ospedale di Circolo in Varese between January 1994 and June 1995 were investigated cytogenetically. Solid tumour samples were obtained from the patients at the time of their initial laparatomy. All patients were newly diagnosed as having previously untreated epithelial endometrial cancer.

The surgically removed specimens were sent under sterile conditions for histological and cytogenetic investigations. Sampling for histopathological and cytogenetic studies was performed in contiguous areas and from a non-necrotic portion of the primary endometrial carcinomas.

The clinical and histological characteristics of the tumours studied, of both type I and type II, are summarized in Table 1. Staging was established according to the FIGO guidelines (Creasman, 1989).

### Histological study

After formalin fixation and paraffin embedding, haematoxylin- and eosin-stained tumours were classified according to the criteria of the WHO (Scully et al, 1994). Malignant neoplasms were subdivided into well (G1), moderately (G2) and poorly differentiated (G3). The grade (G) was based on both nuclear and architectural features as recommended by the FIGO Staging System (Creasman, 1989) and WHO (Scully et al, 1994). The mitotic index of each tumour was estimated on ten high-power fields (HPF) at a magnification of 400 ×.

### Cytogenetics study

Chromosome analysis was performed in each case on direct preparations using the method reported by Dalprà et al (1986), with some

**Table 1** Histopathological aspects of endometrial cancers

| Case no. | Age | Clinical subtype | Histotype | G | Mitotic index (10 × HPF) | Stage |
|---|---|---|---|---|---|---|
| 1 | 49 | I | Endometrioid | 2 | NE | IC |
| 2 | 65 | I | Endometrioid | 1 | 1 | IC |
| 3 | 60 | I | Endometrioid | 1 | 0[a] | IB |
| 4 | 62 | I | Endometrioid | 3 | 22 | IB |
| 5 | 56 | I | Endometrioid | 2 | 10 | IC |
| 6 | 57 | I | Endometrioid | 2 | 22 | IC |
| 7 | 71 | I | Endometrioid with squamous differentiation | 2 | 3 | 3C |
| 8 | 67 | I | Endometrioid with squamous differentiation | 3 | 6 | IC |
| 9 | 84 | I | Endometrioid | 2 | 9 | IC |
| 10 | 62 | I | Endometrioid | 2 | 3 | IB |
| 11 | 84 | I | Endometrioid | 2 | 17 | IB |
| 12 | 87 | – | Mixed mullerian | 3 | 80 | IC |
| 13 | 69 | II | Serous papillary | 2 | 0 | IB |
| 14 | 73 | II | Serous papillary | 3 | 11 | IB |
| 15 | 68 | II | Undifferentiated | 3 | 14 | IB |

[a]Evaluated on 2 × HPF only. NE, not evaluable.

**Table 2** Cytogenetic and FISH results

| Case no. | Histotype | G | Karyotype | FISH analysis |
|---|---|---|---|---|
| 1 | Endometrioid | 2 | 46,XX[5] | Not performed |
| 2 | Endometrioid | 1 | 46,XX [8] | Normal chromosome 6 confirmed |
| 3 | Endometrioid | 1 | 45, XX, –18 [3]/46,XX [8] | Normal chromosome 6 confirmed |
| 4 | Endometrioid | 3 | 46,XX, del(6)(q24–qter) [3]/46,XX [3] | 6q deletion confirmed |
| 5 | Endometrioid | 2 | 46,XX,del(17)(p12–pter) [3]/46,XX [3] | Not performed |
| 6 | Endometrioid | 2 | 46,XX, del(6)(q25–qter) [6]/46,XX [5] | 6q deletion confirmed |
| 7 | Endometrioid with squamous differentiation | 2 | 40–45,XX, +1 [2], – 19 [4] [cp5] | Gain of chromosome 1 confirmed |
| 8 | Endometrioid with squamous differentiation | 3 | 43–45,XX, del(6) (q25–qter)[4], t(9;11)[4][cp7]/46,XX[4] | Not available |
| 9 | Endometrioid | 2 | 34–44,XX,–X [3], –13 [3], –15 [5], – 20 [3], – 21 [5], – 22[3] [cp6] | Normal chromosome 6 confirmed |
| 10 | Endometrioid | 2 | 39–46,XX,–6 [3], del(6) (q21–qter) [5], –15 [3] [cp7] | Not performed |
| 11 | Endometrioid | 2 | 46–48,XX, + 1 [6], del(6) (q25–qter) [4], –8 [3], –9 [4], +11 [2], –12 [3] [cp6] | Gain of chromosome 1 confirmed |
| 12 | Mixed müllerian | 3 | 46–56, XX, t(1;?) [8], + 2[4], + 3 [3], –6 [3], del (6)(q24–qter) [3], – 12 [3], add(12)(p?) [3], – 16 [3], – 18 [7], – 19 [6], – 20 [5] [cp8] | Translocation of chromosome 1 and 6q deletion confirmed |
| 13 | Serous papillary | 2 | 38–46,XX, – 18 [3], del(6) (q25–qter) [7] [cp7]/17–32,X,+6 [3], –7 [2], – 8 [4], – 10 [3], + 14 [3], – 15 [2], – 17 [3], +20 [3] [cp5] | 6q deletion confirmed |
| 14 | Serous papillary | 3 | Not analysable | Chromosome 6 fragmentation confirmed |
| 15 | Undifferentiated | 3 | Not analysable | Chromosome 6 fragmentation confirmed |

modifications. Suspensions of tumour cells were obtained by mincing small pieces of the tumour in a Petri dish and incubated for 72 h at 37°C with 5% carbon dioxide. The medium used was RPMI-1640 supplemented with 15% fetal calf serum, 1% penicillin and streptomycin, 1% L-glutamine, insulin (1 μg $ml^{-1}$), cholera toxin (100 ng $ml^{-1}$) and epidermal growth factor (1 ng $ml^{-1}$) (Pejovic et al, 1989). The tumour cells were exposed overnight to colcemid (0.02 μg $ml^{-1}$) and harvested by hypotonic treatment in 1% sodium citrate and repeated fixations in methanol–acetic acid (3:1). The cell suspension was obtained using a solution of 70% acetic acid and the metaphase spread was performed on a warm plate at 40°C. Karyotype analysis was performed using the QFQ banding technique (ISCN, 1975). A minimum of five metaphases (generally ten) were analysed. Structural abnormalities were identified as clonal if found in two or more cells. Numerical changes (two or more cells for gain, three or more cells for loss) were described relative to the ploidy of the abnormal modal population, as recommended (ISCN, 1995). When different tumour cell populations were identified the modal chromosome number of each population was reported.

### Probes

Four types of probes were used: a biotin-labelled whole chromosome painting (WCP) for chromosome 1, a digoxigenin-labelled WCP for chromosome 6 (ONCOR) and two yeast artificial chromosome (YAC) clones mapped in 6q26–27 (ICRF 17AI12 and 74E9; R Taramelli in preparation). These YAC clones are located in a region approximately 2 cM between markers D6S149 and

 © Cancer Research Campaign 1997

**Figure 1** Endometrial adenocarcinoma (case 4). (**A**) Histological features, complex proliferation of crowded neoplastic glands lined by tall columnar epithelium (H-EX400). (**B**) QFQ-banded metaphase of case 4 showing 6q deletion. (**C**) FISH with WCP for chromosome 6 showing different sizes of two homologues

D6S193. YAC (DNA) probes were labelled with biotinylated 16-dUTP (Boehringer) using the random priming technique.

### Fluorescence in situ hybridization (FISH)

FISH, using WCP of chromosome 6 or WCP of chromosome 1 and YACs, was performed following the method of Pinkel et al (1986)

**Figure 2** Serous papillary endometrial carcinoma (case 13). (**A**) Histological aspect showing papillary pattern of growth and characteristic hobnail-shaped cells (H-EX400). (**B**) QFQ-banded metaphase of diploid cell line of case 13 showing 6q deletion and monosomy of chromosome 18. (**C**) Dual-colour FISH showing loss of YACs mapped in the 6q27 region in the diploid cell line with modifications. Slides were treated with RNAase A (100 μg ml$^{-1}$, Sigma) for 1 h at 37°C, washed twice in 2 × SSC and dehydrated through 70%, 95% and 100% ethanol. The chromosomal DNA was denaturated in 70% formamide, 2 × SSC, pH 7.0, at 75°C for 5 min,

© Cancer Research Campaign 1997

dehydrated in an ice-cold ethanol series and air dried. Cot-1 and YAC DNA mixed with chromosome 6 paint were denaturated at 80°C for 10 min and preannealed for 2 h at 37°C. Hybridization was carried out at 37°C overnight in a humid chamber.

Slides were washed three times in 50% formamide at 42°C, 2 × SSC, pH 7.0, and three times in 2 × SSC at 42°C. Hybridized probes were detected by incubating slides at 37°C in a mixture of rhodamine–antidigoxigenin (ONCOR) and fluorescein–avidin DCS (Vector Laboratories). The amplification step was performed with rabbit anti-sheep and anti-rabbit antibodies (ONCOR) for the digoxigenin-labelled probes, and anti-avidin antibody (Vector) for the biotin-labelled probes. After incubation, slides were washed three times in 4 × SSC, 0.05% Tween 20, and then dehydrated through an ethanol series. Finally, preparations were mounted in an antifade solution containing DAPI or propidium iodide and observed with a Leica DMR fluorescence microscope under a triple-bandpass or FITC filter.

### RESULTS

### Conventional cytogenetic analysis

Conventional cytogenetic analyses of tumours from all patients are summarized in Table 2. Clonal chromosome aberrations were found in 11 endometrial cancers, whereas two cancers displayed normal karyotypes. The modal chromosome number was in the diploid range in all tumours with an abnormal karyotype. In four cases normal clones were detected together with clones with a single anomaly (cases 3, 4, 5 and 6). Two carcinomas (cases 14 and 15) shared chromosome instability and the karyotypes were not analysable.

In seven cases (7, 8, 9, 10, 11, 12, 13) composite karyotypes were observed and in two of these (cases 8 and 13) two different clones were shown.

Chromosome 1 was involved as numeric and structural anomalies in three cases (7, 11, 12). Loss of the entire chromosome 18 was identified in three cases (3, 12 and 13), one of which (case 3) showed a monosomy of chromosome 18 as the sole anomaly.

The most frequent chromosome abnormality detected in our series of endometrial neoplasms was 6q deletion (7 cases out of 13). This type of aberration was present as a sole anomaly in two cases [cases 4 (Figure 1) and 6] or in association with other types of abnormalities in the remaining five cases (cases 8, 10, 11, 12, 13). The position of the proximal breakpoint varied between bands 6q21 and 6q25, breakpoints at 6q24 and 6q25 being most frequently involved.

### FISH analysis

FISH analysis using WCP for chromosome 1 confirmed the presence of a translocation in case 12 and gain of chromosome 1 in cases 7 and 11.

FISH analysis using WCP for chromosome 6 as probe was performed in eight cases (2, 4, 6, 9, 12, 13, 14, 15), in four of these (cases 4, 6, 12, 13) the loss of part of the long arm of chromosome 6 was confirmed by the presence of two FITC signals of different sizes (Figure 1C). In the remaining two cases (14 and 15) the FITC-conjugated chromosome 6 probe painted different chromosome regions (more than 3 and 4) of small size, suggesting a chromosome fragmentation.

We also performed a dual-colour FISH analysis using simultaneously WCP for chromosome 6 and YACs from the 6q27 region as probes. It was possible to apply this combined analysis to five cases [3, 4, 8, 12, 13 (Figure 2)] and molecular loss of the 6q27 region was detected in three of them (cases 4, 12 and 13). In case 3 normal chromosome 6 was observed and unfortunately we could not evaluate 6q27 molecular loss in case 8 because insufficient metaphases were available.

### DISCUSSION

Our data demonstrate that cytogenetic anomalies are frequently detected in endometrial neoplasms belonging to both type I and type II according to Kurman et al (1994). Eleven of 13 endometrial analysable cancers studied were cytogenetically abnormal. In six cases a mosaic constitution showing clones with normal–aneuploid chromosome constitution (cases 3, 4, 5, 6 and 8) and haploid– near- diploid complement (case 13) were identified. Two endometrial neoplasms had chromosome instability. Cells of these tumours typically contained fragmented chromosomes, quadriradial and/or triradial, and varying complex structural rearrangements preventing complete karyotype descriptions. This chromosomal pattern is typical of ovarian carcinomas (Trent et al, 1985; Thompson et al, 1994), but it has not been described in endometrial cancer.

The catalogue of Mitelman (1994) reported 50 uterine carcinomas, the majority of which showed simple karyotypes. Abnormalities of chromosome 1 and particularly trisomy or tetrasomy of 1q have been described. In addition, abnormalities such as trisomies 2, 7, 10 and 12 have also been found. Although abnormalities of chromosome 1 are reported as recurrent aberrations in endometrial cancers (Couturier et al, 1986; Yoshida et al, 1986; Shah et al, 1994; Bardi et al, 1995), in our study chromosome 1 anomalies were identified in only three cases showing endometrioid and mixed müllerian histotypes. These different results may be partly explained by the different technical approach in the study of chromosomal anomalies. We studied chromosome constitutions of endometrial cancers using direct preparations and it is well known that this technique identifies cells in active proliferation (Dalprà et al, 1986; D'Alessandro et al, 1994; Westphal et al, 1994; Bardi et al, 1995; Rosenberg et al, 1995). We found this technique to be more efficient than short-term cultures in identifying the chromosome abnormalities in cancer cells avoiding those of contaminating tissues.

6q deletion is the cytogenetic abnormality most frequently involved in our cases (7 out of 13 cases), and this anomaly was identified in neoplasms showing endometrioid, mixed müllerian and serous papillary morphological features. It is well known that anomalies of 6q are involved in several human malignancies, and occur at high frequency in serous papillary ovarian carcinomas (Sato et al, 1991; Saito et al, 1992; Foulkes et al, 1993; Mitelman, 1994; Orphanos et al, 1995; Tibiletti et al, 1997). Molecular studies employing loss of heterozygosity (LOH) analysis allowed a region of common deletion to be defined that spans markers D6S149 and D6S193 located in 6q27 (Saito et al, 1992). So far, this chromosome abnormality has not been strictly related to the endometrial carcinomas, although different authors have reported, separately, chromosome 6 deletion in serous papillary, endometrioid and mixed müllerian endometrial cancers (Musilová and Michalová, 1986; Milatovich et al, 1990; Shah et al, 1994; Bardi et al, 1995). We identified a high proportion of endometrial carcinomas showing cytogenetic 6q deletion, and this was confirmed by FISH analysis using YACs from 6q27 as probes. This technique

© Cancer Research Campaign 1997

demonstrated the same allelic loss of a chromosomal region frequently found in ovarian carcinomas.

The finding of cytogenetically normal clones (cases 1, 2, 3, 4, 5, 6 and 8) suggests that more subtle mutations, precluding their assessment by our approach, may be involved in endometrial cancers. The possibility that we analysed the karyotype of non-tumoral cells can be excluded because, on direct preparations of non-tumoral tissues, mitoses were never observed precluding their cytogenetic analysis.

Our findings demonstrate a large heterogeneity in chromosome constitution of endometrial cancer, which may be related to different histological subtypes. The tumour karyotypes of serous papillary (type II cancer) and mixed müllerian were more complex than those of endometrial carcinomas (type I cancer), and this finding may be associated with a less favourable prognosis of the former neoplasm.

On the contrary, no relationships between chromosome constitution and grade, proliferative status and clinicopathological stage were observed, but clearly more cases need to be analysed in order to achieve statistical significance.

The 6q deletion was detected in serous papillary and endometrioid carcinomas of the endometrium that show morphological similarities with the ovarian counterparts. Interestingly, the tumour-suppressor gene(s) mapped to 6q27 by allelotype studies (Sato et al, 1991; Saito et al, 1992; Foulkes et al, 1993), and involved in the pathogenesis of ovarian tumours, might also play a role in endometrial tumours. This is not unexpected given the common histogenesis of müllerian structures.

### ACKNOWLEDGEMENTS

This work was supported by grants from AIRC to RT and from Italian Ministry of Health, Italian University and Research Ministry to CC.

### REFERENCES

Bardi G, Pandis N, Schousboe K, Holund B and Heim S (1995) Near diploid karyotypes with recurrent chromosome abnormalities characterize early-stage endometrial cancers. *Cancer Genet Cytogenet* **80**: 110–114

Bokhman JW (1983) Two pathogenetic types of endometrial carcinoma. *Gynecol Oncol* **15**: 10

Creasman WT (1989) Announcement FIGO stages: 1988 revisions. *Gynecol Oncol* **35**: 125–127

Couturier J, Vielh P, Salmon RJ and Dutrillax (1986) Trisomy and tetrasomy for long arm of chromosome 1 in near-diploid human endometrial adenocarcinomas. *Int J Cancer* **38**: 17–19

D'Alessandro E, Lo Re ML, Crisci R, Ligas C and Coloni GF (1994) Cytogenetic findings in primary non-small cell lung cancer. *Tumori* **80**: 151–156

Dalpra' L, Nocera G, Tibiletti MG, Gramellini F, Agosti S and Oldrini A (1986) Technical aspects and diagnostic problems of direct chromosome analysis using chorionic villus sample in first trimester. *Hum Reprod* **1**(2): 103–106

Foulkes WD, Ragoussis J, Stamp GW, Allan GJ and Trowsdale J (1993) Frequent loss of heterozygosity on chromosome 6 in human ovarian carcinoma. *Br J Cancer* **67**: 551–559

ISCN (1975) Paris Conference Supplement: standardization in human cytogenetics. *Cytogenet Cell Genet* **15**: 201–238

ISCN (1995) *An International System for Human Cytogenetic Nomenclature*. Mitelman F (ed). Karger: Basle

Kurman RJ, Zaino RJ and Norris HJ (1994) Endometrial carcinoma. In *Blaustein's Pathology of the Female Genital Tract*. Kurman RJ (ed), pp. 439–486. Springer: New York

Milatovich A, Heerema NA and Palmer CG (1990) Cytogenetic studies of endometrial malignancies. *Cancer Genet Cytogenet* **46**: 41–54

Mitelman F (1994) *Catalog of Chromosome Aberrations in Cancer*. Johansson B and Mertens F (eds), Wiley-Liss: New York

Musilova' J and Michalova' K (1986) Cytogenetic study of cancer cells in effusions. *Cancer Genet Cytogenet* **19**: 271–279

Orphanos V, McGown G, Hey Y, Thorncroft M, Santibanez-Koref M, Russell SH, Hickey I, Atkinson RJ and Boyle JM (1995) Allelic imbalance of chromosome 6q in ovarian tumors. *Br J Cancer* **71**: 666–669

Pejovic T, Heim S, Mandahl N, Elmfors B, Floderus UM, Fulgyik S, Helm G, Willen H and Mitelman F (1989) Consistent occurrence of a 19p+ marker chromosome and a loss of lip material in ovarian seropapillary cystoadenocarcinomas. *Gene Chromosom Cancer* **1**: 167–171

Pinkel D, Straumet T and Gray JW (1986) Cytogenetic analysis using quantitative, high-sensitivity fluorescence hybridization. *Proc Natl Acad Sci USA* **63**: 2934–2938

Rosenberg C, Della-Rosa VA, Latronico AC, Mendonca BB and Vianna-Morgante AM (1995) Selection of adrenal tumor cell in culture demonstrated by interphase cytogenetics. *Cancer Genet Cytogenet* **79**(1): 36–40

Saito S, Saito H, Koi S, Sagae S, Kudo R, Saito J, Noda K and Nakamura Y (1992) Fine-scale deletion mapping of the distal long arm of chromosome 6 in 70 human ovarian cancers. *Cancer Res* **52**: 5815–5817

Saito T, Saito H, Morita R, Koi S, Lee JH and Nakamura Y (1991) Allelotype of human ovarian cancer. *Cancer Res* **51**: 5118–5122

Scully RE, Bonfiglio TA, Kurman RJ, Silverberg SG and Wikinson EJ (1994) Histological typing of female genital tract tumors. In *WHO International Histological Classification of Tumors*. Scully RE, Poulsen HE and Sobin LH (eds), pp. 439–486 Springer: Berlin

Shah NK, Currie JL, Rosenshein N, Campbell J, Long P, Abbas F and Griffin CA (1994) Cytogenetic and FISH analysis of endometrial carcinoma. *Cancer Genet Cytogenet* **73**: 142–146

Smith M and McCartney AJ (1985) Occult, high-risk endometrial cancer. *Gynecol Oncol* **22**: 154

Thompson FH, Emerson J, Alberts D, Liu Y, Guan XY, Burgess A, Fox S, Teatle R, Weinstein R, Makar R, Powell D and Trent J (1994) Clonal chromosome abnormalities in 54 cases of ovarian carcinoma. *Cancer Genet Cytogenet* **73**: 33–45

Tibiletti MG, Bernasconi B, Furlan D, Riva C, Trubia M, Buraggi G, Franchi M, Bolis PF, Mariani A, Frigerio L, Capella C and Taramelli R (1996) Early involvement of 6q in surface epithelial ovarian tumors. *Cancer Res* **56**: 4493–4498

Trent JM, Thompson F and Buick RN (1985) Generation of clonal variants in a human ovarian carcinoma studies by chromosome banding analysis. *Cancer Genet Cytogenet* **14**: 153–161

Yoshida MA, Ohyashiki K, Piver SM and Sandberg AA (1986) Recurrent endometrial adenocarcinoma with rearrangement of chromosome 1 and 11. *Cancer Genet Cytogenet* **20**: 159–162

Westphal M, Hansel W, Hamel W, Kunzmann R and Holzel F (1994) Karyotype analyses of 20 glioma cell lines. *Acta Neurochir Wien* **126**(1): 17–26

Zanetti R and Crosignani P (1992) Registro tumori Lombardia In *Il Cancro in Italia, I Dati di Incidenza dei Registri Tumori 1983–1987*, Zanetti R and Crosignani P (eds), pp 268–286. Lega Italiana per la lotta contro i tumori: Torino

© Cancer Research Campaign 1997


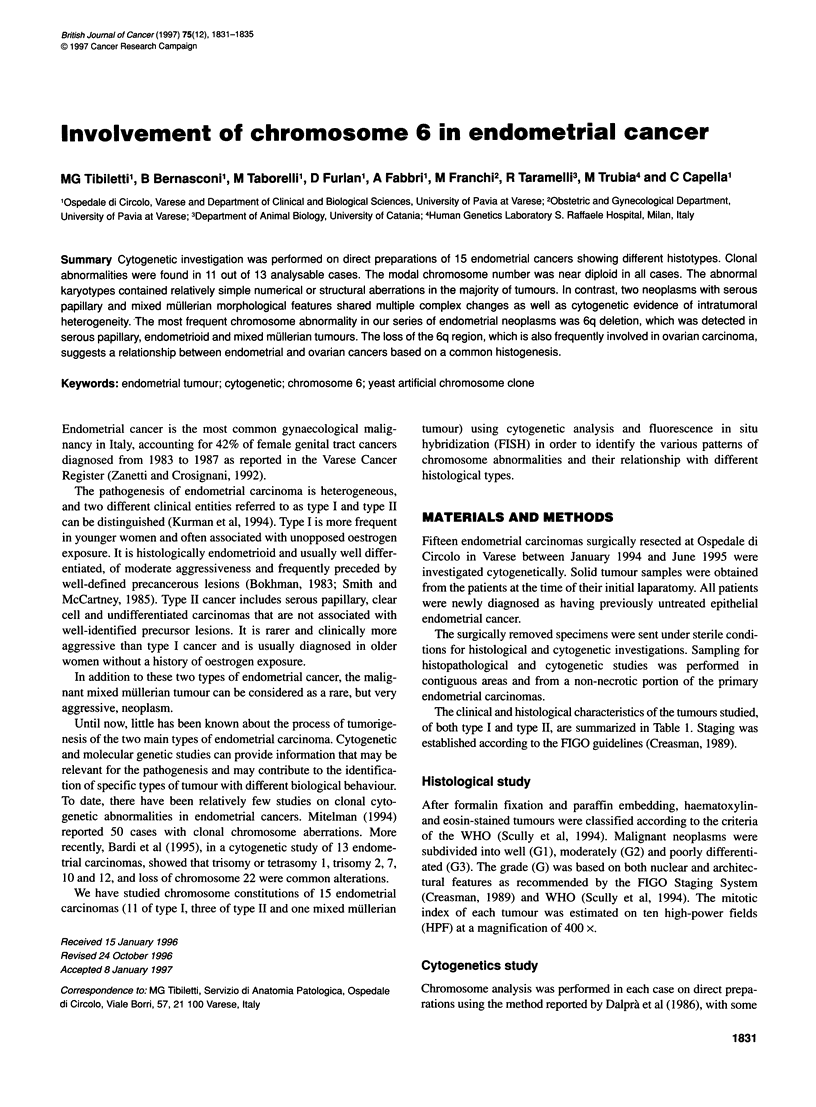

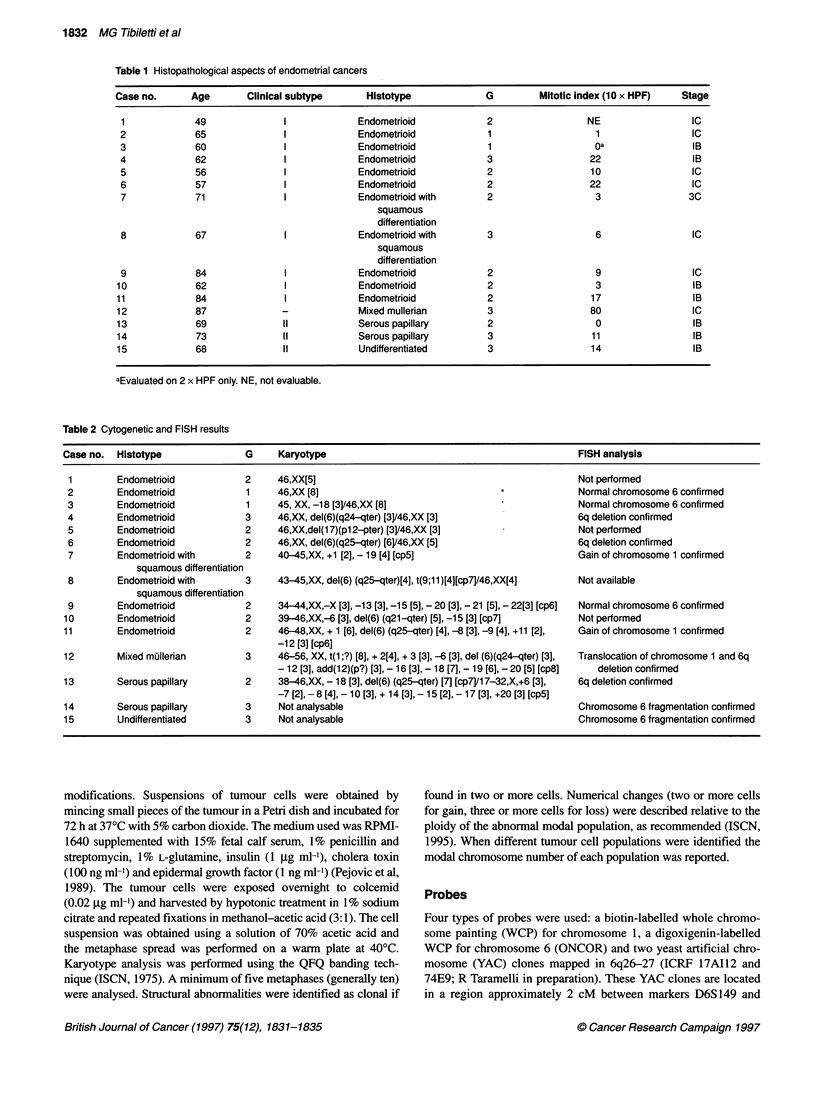

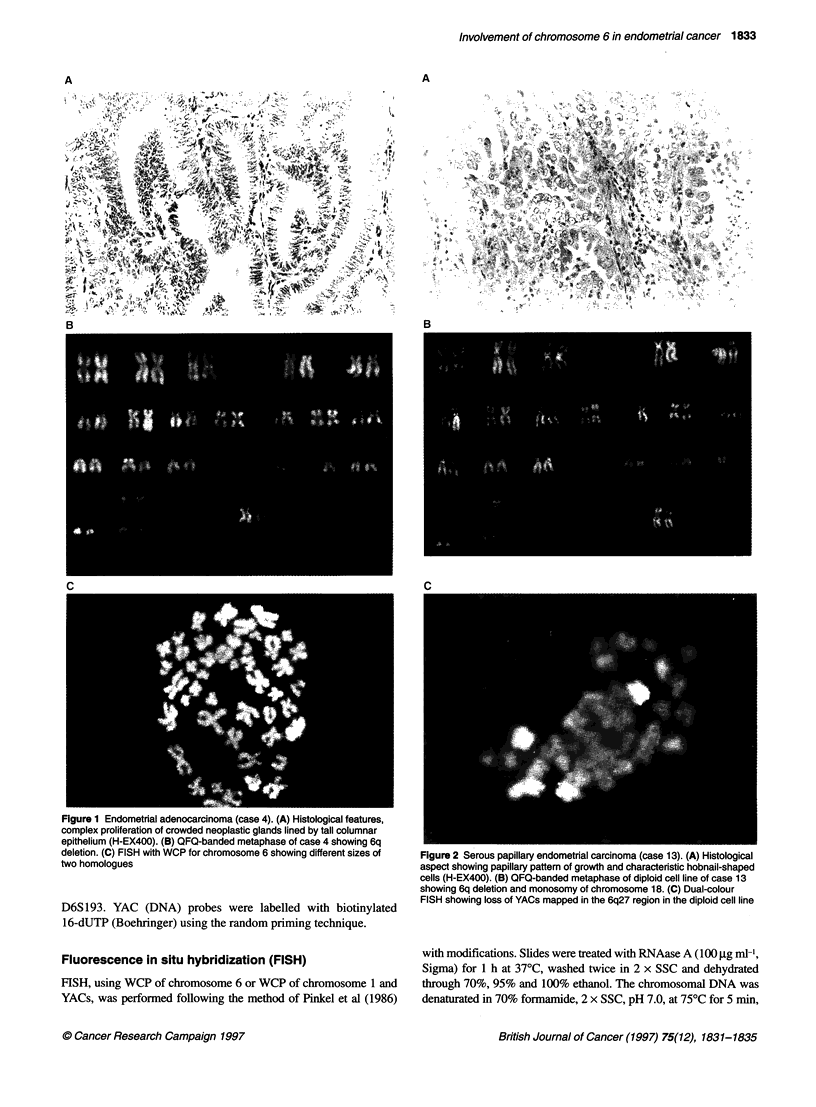

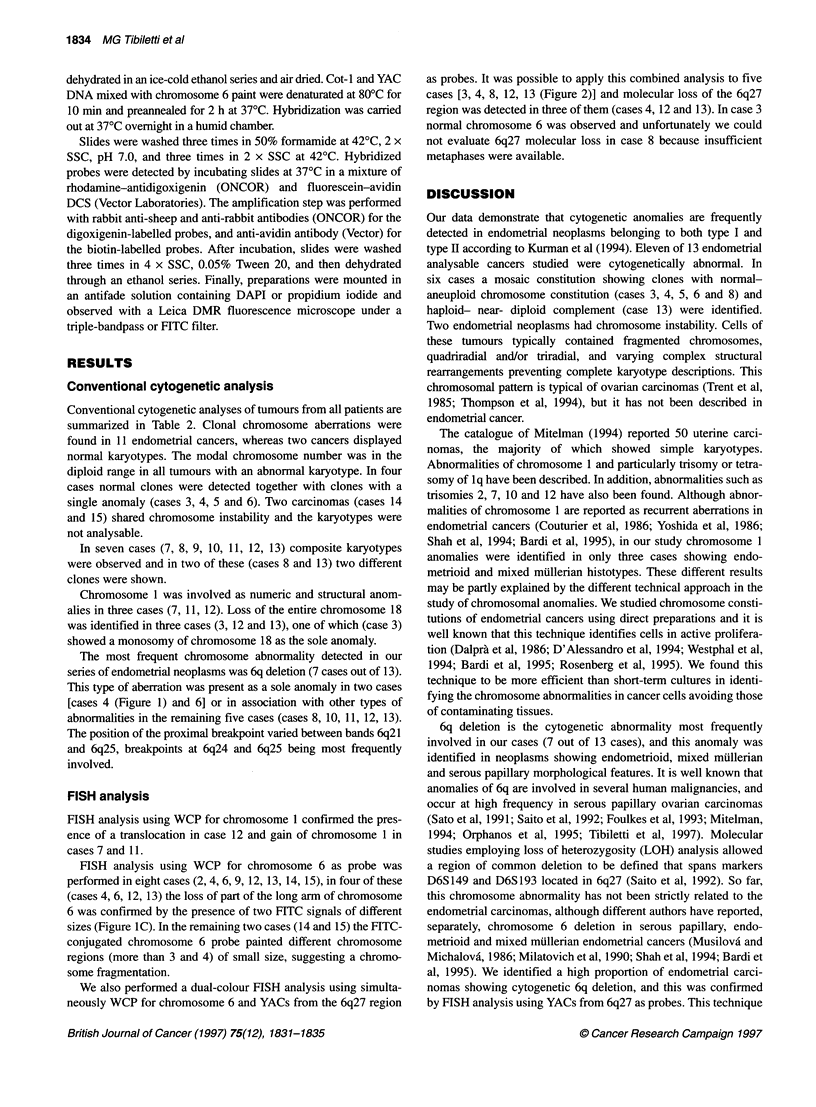

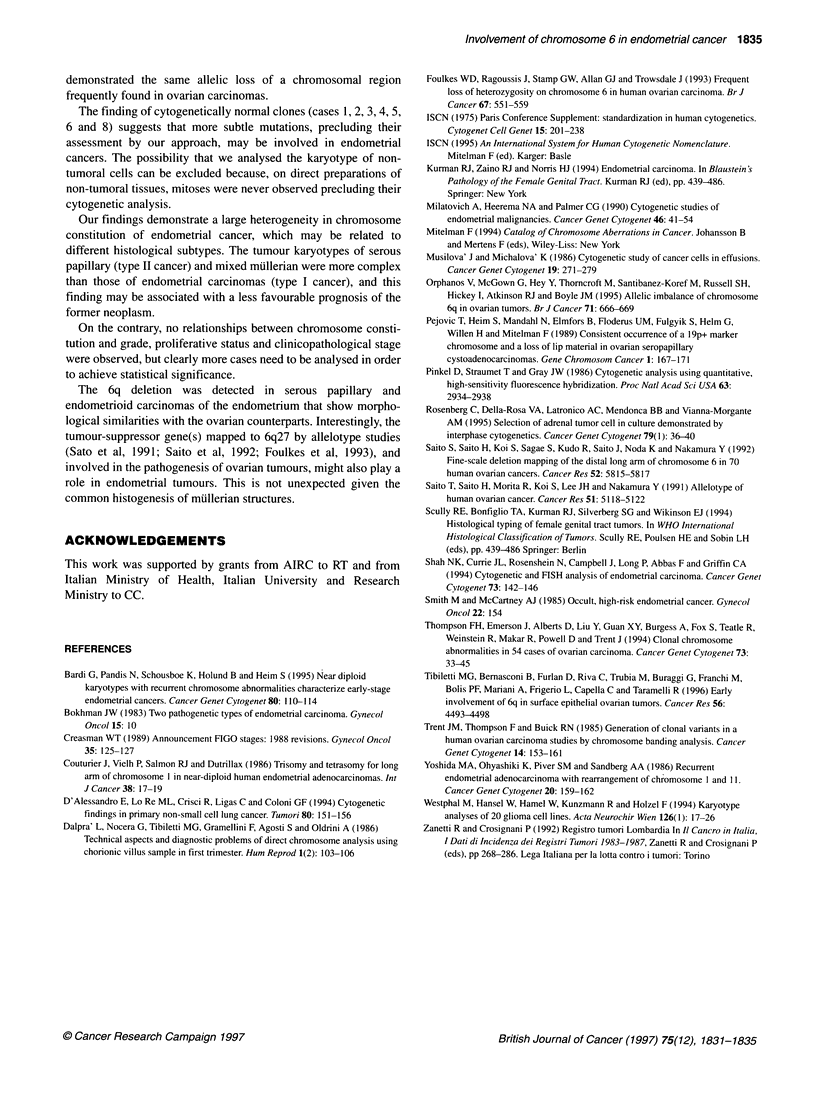


## References

[OCR_00449] Bardi G., Pandis N., Schousboe K., Hølund B., Heim S. (1995). Near-diploid karyotypes with recurrent chromosome abnormalities characterize early-stage endometrial cancer.. Cancer Genet Cytogenet.

[OCR_00454] Bokhman J. V. (1983). Two pathogenetic types of endometrial carcinoma.. Gynecol Oncol.

[OCR_00462] Couturier J., Vielh P., Salmon R., Dutrillaux B. (1986). Trisomy and tetrasomy for long arm of chromosome 1 in near-diploid human endometrial adenocarcinomas.. Int J Cancer.

[OCR_00467] D'Alessandro E., Lo Re M. L., Crisci R., Ligas C., Coloni G. F. (1994). Cytogenetic findings in primary non-small cell lung cancer.. Tumori.

[OCR_00471] Dalprà L., Nocera G., Tibiletti M. G., Gramellini F., Agosti S., Oldrini A. (1986). Technical aspects and diagnostic problems of direct chromosome analysis using chorionic villus sampling in the first trimester.. Hum Reprod.

[OCR_00476] Foulkes W. D., Ragoussis J., Stamp G. W., Allan G. J., Trowsdale J. (1993). Frequent loss of heterozygosity on chromosome 6 in human ovarian carcinoma.. Br J Cancer.

[OCR_00494] Milatovich A., Heerema N. A., Palmer C. G. (1990). Cytogenetic studies of endometrial malignancies.. Cancer Genet Cytogenet.

[OCR_00506] Orphanos V., McGown G., Hey Y., Thorncroft M., Santibanez-Koref M., Russell S. E., Hickey I., Atkinson R. J., Boyle J. M. (1995). Allelic imbalance of chromosome 6q in ovarian tumours.. Br J Cancer.

[OCR_00511] Pejovic T., Heim S., Mandahl N., Elmfors B., Flodérus U. M., Furgyik S., Helm G., Willén H., Mitelman F. (1989). Consistent occurrence of a 19p+ marker chromosome and loss of 11p material in ovarian seropapillary cystadenocarcinomas.. Genes Chromosomes Cancer.

[OCR_00517] Pinkel D., Straume T., Gray J. W. (1986). Cytogenetic analysis using quantitative, high-sensitivity, fluorescence hybridization.. Proc Natl Acad Sci U S A.

[OCR_00522] Rosenberg C., Della-Rosa V. A., Latronico A. C., Mendonça B. B., Vianna-Morgante A. M. (1995). Selection of adrenal tumor cells in culture demonstrated by interphase cytogenetics.. Cancer Genet Cytogenet.

[OCR_00527] Saito S., Saito H., Koi S., Sagae S., Kudo R., Saito J., Noda K., Nakamura Y. (1992). Fine-scale deletion mapping of the distal long arm of chromosome 6 in 70 human ovarian cancers.. Cancer Res.

[OCR_00532] Sato T., Saito H., Morita R., Koi S., Lee J. H., Nakamura Y. (1991). Allelotype of human ovarian cancer.. Cancer Res.

[OCR_00543] Shah N. K., Currie J. L., Rosenshein N., Campbell J., Long P., Abbas F., Griffin C. A. (1994). Cytogenetic and FISH analysis of endometrial carcinoma.. Cancer Genet Cytogenet.

[OCR_00548] Smith M., McCartney A. J. (1985). Occult, high-risk endometrial cancer.. Gynecol Oncol.

[OCR_00552] Thompson F. H., Emerson J., Alberts D., Liu Y., Guan X. Y., Burgess A., Fox S., Taetle R., Weinstein R., Makar R. (1994). Clonal chromosome abnormalities in 54 cases of ovarian carcinoma.. Cancer Genet Cytogenet.

[OCR_00559] Tibiletti M. G., Bernasconi B., Furlan D., Riva C., Trubia M., Buraggi G., Franchi M., Bolis P., Mariani A., Frigerio L. (1996). Early involvement of 6q in surface epithelial ovarian tumors.. Cancer Res.

[OCR_00565] Trent J. M., Thompson F. H., Buick R. N. (1985). Generation of clonal variants in a human ovarian carcinoma studied by chromosome banding analysis.. Cancer Genet Cytogenet.

[OCR_00575] Westphal M., Hänsel M., Hamel W., Kunzmann R., Hölzel F. (1994). Karyotype analyses of 20 human glioma cell lines.. Acta Neurochir (Wien).

[OCR_00570] Yoshida M. A., Ohyashiki K., Piver S. M., Sandberg A. A. (1986). Recurrent endometrial adenocarcinoma with rearrangement of chromosomes 1 and 11.. Cancer Genet Cytogenet.

